# Mother’s Milk Microbiome Shaping Fecal and Skin Microbiota in Infants with Food Allergy and Atopic Dermatitis: A Pilot Analysis

**DOI:** 10.3390/nu13103600

**Published:** 2021-10-14

**Authors:** Marcin Gołębiewski, Ewa Łoś-Rycharska, Marcin Sikora, Tomasz Grzybowski, Marta Gorzkiewicz, Aneta Krogulska

**Affiliations:** 1 Department of Plant Physiology and Biotechnology, Nicolaus Copernicus University, 87-100 Torun, Poland; 2 Interdisciplinary Centre of Modern Technologies, Nicolaus Copernicus University, 87-100 Torun, Poland; sikoramar@umk.pl; 3 Department of Pediatrics, Allergology and Gastroenterology, Collegium Medicum Bydgoszcz, Nicolaus Copernicus University, 87-100 Torun, Poland; aneta.krogulska@cm.umk.pl; 4 Department of Forensic Medicine, Collegium Medicum Bydgoszcz, Nicolaus Copernicus University, 87-100 Torun, Poland; tgrzyb@cm.umk.pl (T.G.); gorzkiewiczmarta@cm.umk.pl (M.G.)

**Keywords:** atopic dermatitis, dysbacteriosis/microbial imbalance, food allergy, feces, mother’s milk, microbiota, skin, 16S rRNA sequencing

## Abstract

The child microbiome, including gut and skin communities, is shaped by a multitude of factors, and breastfeeding is one of the most essential. Food allergy (FA) and atopic dermatitis (AD) are among the most common diseases in pediatrics, with the prevalence of each up to 6% and 20%, respectively. Therefore, we aimed at finding differences between the fecal and skin microbiomes of FA and AD patients in the context of breastfeeding, by means of the Illumina sequencing of 16S rRNA gene fragment libraries amplified from the total DNA isolated from samples collected from allergic and healthy infants. We also analyzed milk samples from the mothers of the examined children and searched for patterns of incidence suggesting milk influence on an infant’s allergy status. Here we show that a mother’s milk influences her child’s fecal and skin microbiomes and identify *Acinetobacter* as the taxon whose abundance is correlated with milk and child-derived samples. We demonstrate that breastfeeding makes allergic children's fecal and skin communities more similar to those of healthy infants than in the case of formula-feeding. We also identify signature taxa that might be important in maintaining health or allergy development.

## 1. Introduction

The child microbiome, including gut and skin communities, is shaped by a multitude of factors [1,2,3,4]. Breastfeeding is one of the most important ones, modulating the gut microbiome in particular [2,3,5,6,7,8,9,10,11,12]. Its effects exerted on the gut microbiome last beyond infancy [5,13,14]. Differences between the fecal microbiome of breastfed and formula-fed children have been demonstrated in many studies, e.g., [2,5,15]. These differences are caused not only by the mother’s milk microbiome but also by other compounds present in milk that are either nutrients for probiotic bacteria (i.e., prebiotics), such as non-digestible oligosaccharides (HMO—human milk oligosaccharides [6,15]) or immunomodulating compounds, e.g., TGF-beta2 [7]. It was shown that even partial breastfeeding was the most influential factor in a child’s gut microbiome development [16]. Moreover, it was found that changes in children’s feces microbiome during weaning were caused to a lesser degree by the newly introduced food and more by the cessation of breastfeeding [17].

The direct transmission of certain microbes from the mother’s milk to her child’s gut has been demonstrated [2,8,11,18,19]. Interestingly, breastfeeding by a child’s own mother and by other women affects the child’s feces microbiome in different ways [20]. Infant feces analysis demonstrated the transfer of orally administered probiotic bacteria from mother to child [21]. It was also found that the microbiomes of different body parts influenced each other and that surrounding microbiomes directly shaped a child’s microbial community, particularly on the skin [1,2,4].

Data on the relation of the mother’s milk microbiome (and even the very fact of breastfeeding) and her child’s skin microbial community are scarce—there is only one study focused on *Staphylococcus* transfer from the mother to the child’s skin and gut [22]. There is no data on bacteria coincidence in the mother’s milk and her child’s body compartments other than guts. Breastfeeding may decrease the negative influence of diseases on a child’s gut microbiome, e.g., lower the risk of dysbiosis caused by diarrhea [5] or by prematurity and immature gut [23]. Moreover, breastfeeding causes the fecal microbiome of children delivered by cesarean section to be more similar to that of children naturally delivered than in the case of formula-fed ones [6,13,24]. The mother’s milk may be a factor in decreasing the probability and halting the development of certain conditions, such as allergy [8] or obesity [25]. On the other hand, it was demonstrated that the transfer of certain bacteria from milk to the child’s guts might be detrimental. Milk-transferred bacteria such as *Methylobacterium komagatae*, *Methylocapsa palsarum* and *Bacteroides vulgatus* are possibly related to an increased probability of developing celiac disease in childhood [19]. Similarly, the involvement of Egerthellaceae members [18] and lower microbiome diversity in the mother's milk [26] in allergy development was implied.

Breastfeeding-induced changes in the bacterial community structure of children's guts leading to atopic dermatitis (AD) development cannot be excluded [17]. There were differences in the metabolic profile of the gut microbiome in infants suffering from AD depending on the feeding mode. Genes involved in oxidative phosphorylation were more frequent in breastfed AD children than in healthy ones. Regardless of the feeding mode, the fecal communities of AD children were more diverse than those in healthy subjects, but the number of microbial cells was lower [27]. Feeding infants with partially hydrolyzed milk protein mixture enriched with a prebiotic was shown to make their gut microbiome more similar to that of breastfed children, both with regards to taxonomic composition and the metabolic profile of the community [9]. These facts were suggested to be related to a decreased risk of AD development until 18 months of age in children from a risk group [9].

The objective of our study was to compare mothers’ milk microbiomes with the fecal and skin microbiomes of their children to find out if there were patterns depending on the presence of allergy and the mode of feeding. Moreover, we wanted to check if there were OTUs (Operational Taxonomic Units), whose abundance was correlated in milk, feces and skin, depending on the feeding mode and the presence of allergy. As far as we know, this is the first study of this kind. To achieve these goals, we generated and sequenced V3-V4 16S rRNA gene libraries and analyzed the resulting sequences.

## 2. Materials and Methods

This is a part of stage I of a two-stage prospective study, hence the number of subjects is relatively low and there are missing data. As the latter only impact correlations, we decided to use the data in spite of their shortcomings.

### 2.1. Study Group

**Figure 1:**
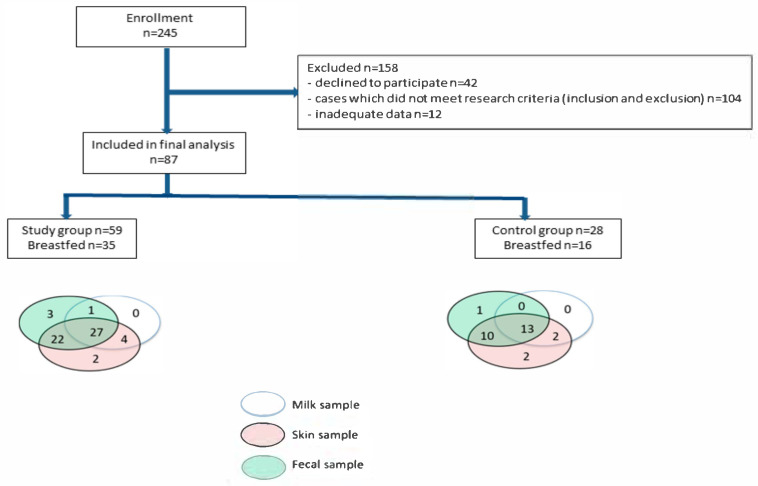
Study design. Diagram showing steps involved in patient selection and samples tests collected.

The study group was described in detail earlier [28]. A diagram depicting the steps of patients' enrollment and the samples collected in this study is shown in Figure 1.

### 2.2. Sample Collection

The methods of the fecal and skin samples collection were reported earlier [28]. Milk samples were taken during the enrollment visit with sterile, disposable equipment, and freshly expressed mothers' milk was gathered into collection tubes and frozen at −80 °C until further processing.

### 2.3. Metagenomic Analysis

DNA was isolated and Illumina sequencing libraries generation was carried out as previously described [28,29]. The libraries were sequenced on MiSeq using 600 cycles v.3 kit (Illumina) at CMIT NCU.

### 2.4. Bioinformatics and Statistical Analyses

The process of sequencing reads analysis was described earlier [28,29]. Briefly, it was performed in R with the use of functions from the dada2 package [30] and Silva v. 132 [31]. The sequences were then clustered into OTUs (i.e., clusters of sequences more than 97% similar, roughly corresponding to bacterial species), and shared OTU tables were computed with Mothur [32]. Ecological analyses were carried out in R using functions from the vegan [33] and GuniFrac packages [34,35]. Unweighted UniFrac distances were calculated from a rarefied OTU table and a tree (computed with the Relaxed Neighbor Joining [36]) using the GUniFrac function. Non-metric multidimensional scaling (NMDS) implemented in the metaMDS function was used to perform unconstrained ordinations. PERMANOVA (adonis function; 999 permutations) served as a test for grouping significance, dbRDA on distance matrix was computed using dbrda, and model significance was tested with anova.rda (999 permutations). *p*-value < 0.05 was regarded as significant. Automated stepwise model construction was carried out using the ordistep function using direction = both and 999 permutations.

Sparse partial least squares discriminant analysis (sPLS-DA) was carried out using splsda function from the mixOmics v. 6.14.0 package [37,38] according to the protocol described at http://mixomics.org/case-studies/splsda-srbct/ (accessed on 2 June 2021).

Differential abundance was tested using ANOVA (aov function in R); homogeneity of variance was assessed by Levene’s test (levene.test of the lawstat package), and the normality of data with the Shapiro–Wilk test (shapiro.test). In case the assumptions were violated, the Kruskal–Wallis test was used (kruskal.test). Benjamini–Hochberg FDR (p.adjust) was used as a correction for multiple comparisons; a significance level of 0.05 was used. A two-tailed Fisher’s exact test in R (fisher.test) was used to analyze clinical categorical data; again, a significance level of 0.05 was used. Correlations between clinical variables and alpha diversity, as well as between OTUs abundance in different compartments, were tested using Spearman’s rho.

### 2.5. Additional Information

All methods were carried out in accordance with the relevant guidelines and regulations. The research was conducted with the consent of the local Ethics Committees of the Institutional Review Board of CM Bydgoszcz NCU Torun, Poland (765/2017). Written informed consent was obtained from the parents of each patient prior to their study enrollment.

## 3. Results

### 3.1. Alpha Diversity

**Figure 2:**
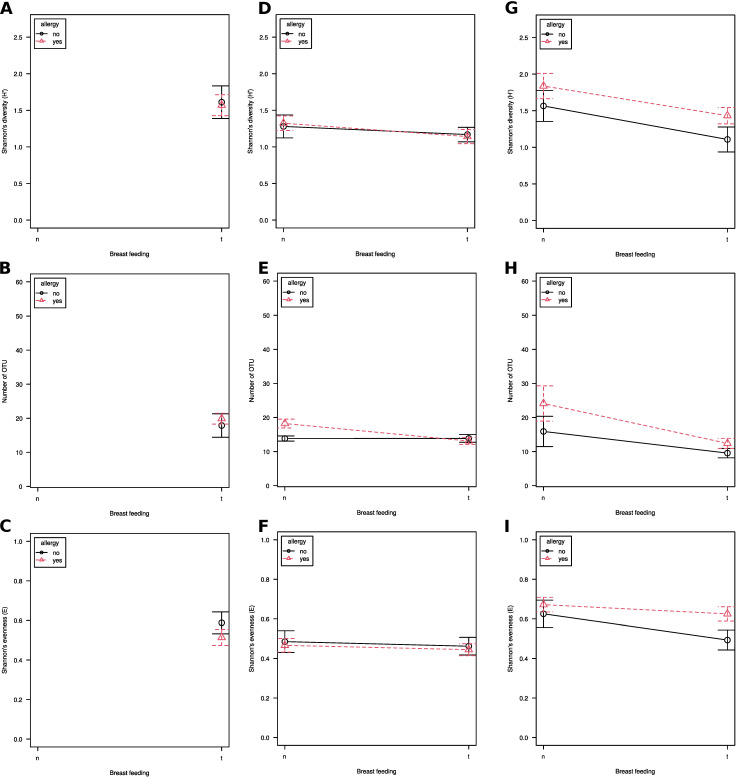
Bacterial communities alpha diversity (Shannon’s H’; panels (**C**,**F**,**I**)), species richness (number of observed OTUs; panels (**B**,**E**,**H**)) and evenness (Shannon’s E; panels (**A**,**D**,**G**)) in milk (panels (**A**–**C**)), feces (panels (**D**–**F**)) and skin (panels (**G**–**I**)) samples. Whiskers denote 95% CI.

Milk samples from mothers of allergic and healthy children did not differ in diversity (Shannon’s H’), species richness (number of OTUs) or evenness (Shannon’s E) (Figure 2A–C). Diversity and evenness in breastfed and formula-fed children's feces were similar, regardless of their allergic status (Figure 2D,F), while species richness was significantly lower in breastfed ones (*p* = 0.005; Figure 2E). When patients were grouped according to clinical status, lower species richness was observed in the fecal communities of breastfed allergic children than in formula-fed allergic ones (*p* = 0.04) and was similar to species richness of healthy ones, while there was no difference in healthy children (*p* > 0.05).

Skin microbiome diversity was significantly lower in breastfed children, regardless of their clinical status (*p* = 0.01). The same applied to species richness (*p* = 0.004; Figure 2G,H), while there were no differences in evenness (*p* > 0.05; Figure 2I). No clinical variable significantly correlated with alpha diversity, regardless of the group assessed.

### 3.2. Beta-Diversity

**Figure 3:**
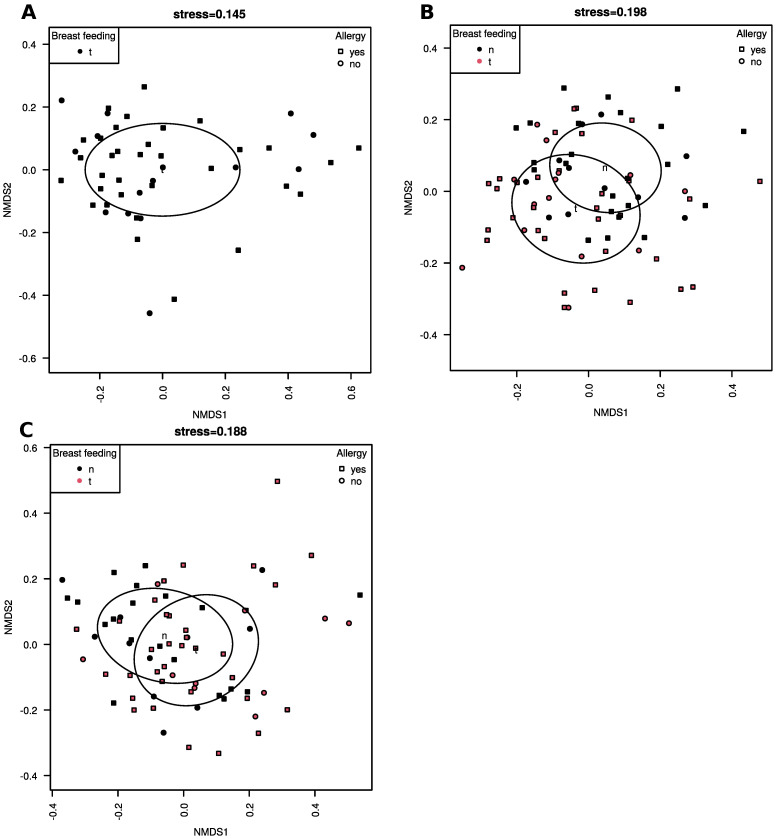
NMDS ordinations of unweighted UniFrac distance matrices derived from (**A**) milk, (**B**) feces and (**C**) skin communities. Ellipses show 95% CI.

Milk microbiome community structure did not differ significantly in allergic and healthy children (PERMANOVA, *p* > 0.05; Figure 3A–C). There were significant differences in the community structure between breastfed and formula-fed children in fecal (PERMANOVA, *p* = 0.001; Figure 3B) and skin (PERMANOVA, *p* = 0.003; Figure 3C) samples, however, there were no differences according to allergic status. No clinical variables were found to correlate significantly with community structure in any material or clinical group (dbRDA, *p* > 0.05).

### 3.3. Taxonomic Composition

**Figure 4:**
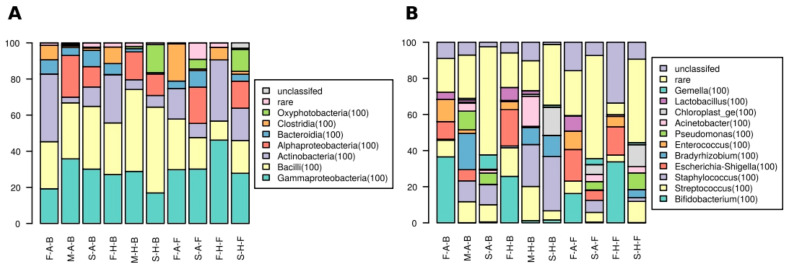
Bacterial communities taxonomic composition at (**A**) class and (**B**) genus levels. Taxa with an overall share below 2% are shown as ‘rare’ category. Sample types encoding: first letter—compartment (F—feces, M—milk, S—skin), second letter: allergic status (A—allergy, H—healthy control), third letter: feeding mode (B—breastfed, F—formula-fed).

At the level of class Gammaproteobacteria, Bacilli and Actinobacteria prevailed, while Alphaproteobacteria, Bacteroidia, Clostridia and Oxyphotobacteria abundance was lower (Figure 4A). There were no statistically significant differences in taxa abundance, regardless of the groups compared.

Bacilli were more abundant in the milk samples from the mothers of healthy infants, while the opposite was true for Alphaproteobacteria, albeit the differences were inconspicuous.

Gammaproteobacteria were less abundant in the fecal samples of breastfed children than in formula-fed ones, and within each of these groups, the abundance was lower in the feces of allergic patients. Actinobacteria were more abundant in breastfed allergic children’s feces than in the fecal samples from healthy ones, while the abundance in allergic ones was lower in the formula-fed patients’ feces. Abundance of Clostridia and Bacilli was similar in the feces of breastfed children, regardless of their clinical status, while in formula-fed samples, they were more abundant in allergic ones.

Libraries generated from skin samples were the only ones where Oxyphotobacteria were found; the reads were affiliated with *Chloroplast*. They were more abundant in samples from healthy children than in allergic ones.

At the level of genus (Figure 4B) *Bifidobacterium*, *Enterococcus* and *Lactobacillus* were characteristic for fecal samples. *Bifidobacteirum* was more abundant in libraries from allergic breastfed and healthy formula-fed children. *Enterococcus* was more abundant in allergic children than in healthy ones, regardless of the mode of feeding, while *Lactobacillus* was more frequent in healthy children in the groups of breastfed patients and in formula-fed ones it was more numerous in allergic ones.

There were no genera present solely in the milk samples; all were shared with fecal or skin ones. Libraries derived from the milk of allergic infants’ mothers contained a large number of *Bradyrhizobium*, *Escherichia*-*Shigella* and *Pseudomonas* reads. The milk of healthy children’s mothers was characterized by large shares of *Staphylococcus* and *Streptococcus*, as well as *Acinetobacter*.

### 3.4. Significant Differences in Taxa Abundance

A comparison of bacterial taxa abundance between the samples from healthy and allergic children and their mothers showed that significant differences were present only at the OTU level (Table 1). OTU3 (*Pseudomonas peli*), OTU40 (*Bacteroides ovatus*) and OTU93 (*Leptotrichia wadei*) were significantly more abundant in the milk of healthy children’s mothers, and the two first OTUs were absent from the milk samples of allergic children’s mothers. OTU30 (*Serratia marcescens*) and OTU68 (*Lactococcus lactis*) were more frequent in fecal samples from healthy infants, while OTU35 (Parabacteroides sp.) was more abundant in the feces of allergic ones. There were no differentially abundant OTUs in the skin samples.

Libraries from fecal samples of allergic formula-fed children were characterized by a greater abundance of reads classified as *Blautia* and *Escherichia-Shigella*, and, at the OTU level, of OTU1 (*Escherichia-Shigella*), OTU52 (Peptostreptococcaceae) and OTU55 (Lachnospiraceae). Skin samples from formula-fed allergic patients were more abundant in orders Enterobacteriales and Pseudomonadales and families Enterobacteriaceae and Moraxellaceae.

In the group of healthy children, OTU23 (*Blautia*) was more frequent in fecal samples, and reads affiliated with Bacteroidales were more abundant on the skin of formula-fed infants.

### 3.5. Identification of Signature Taxa (sPLS-DA)

**Figure 5:**
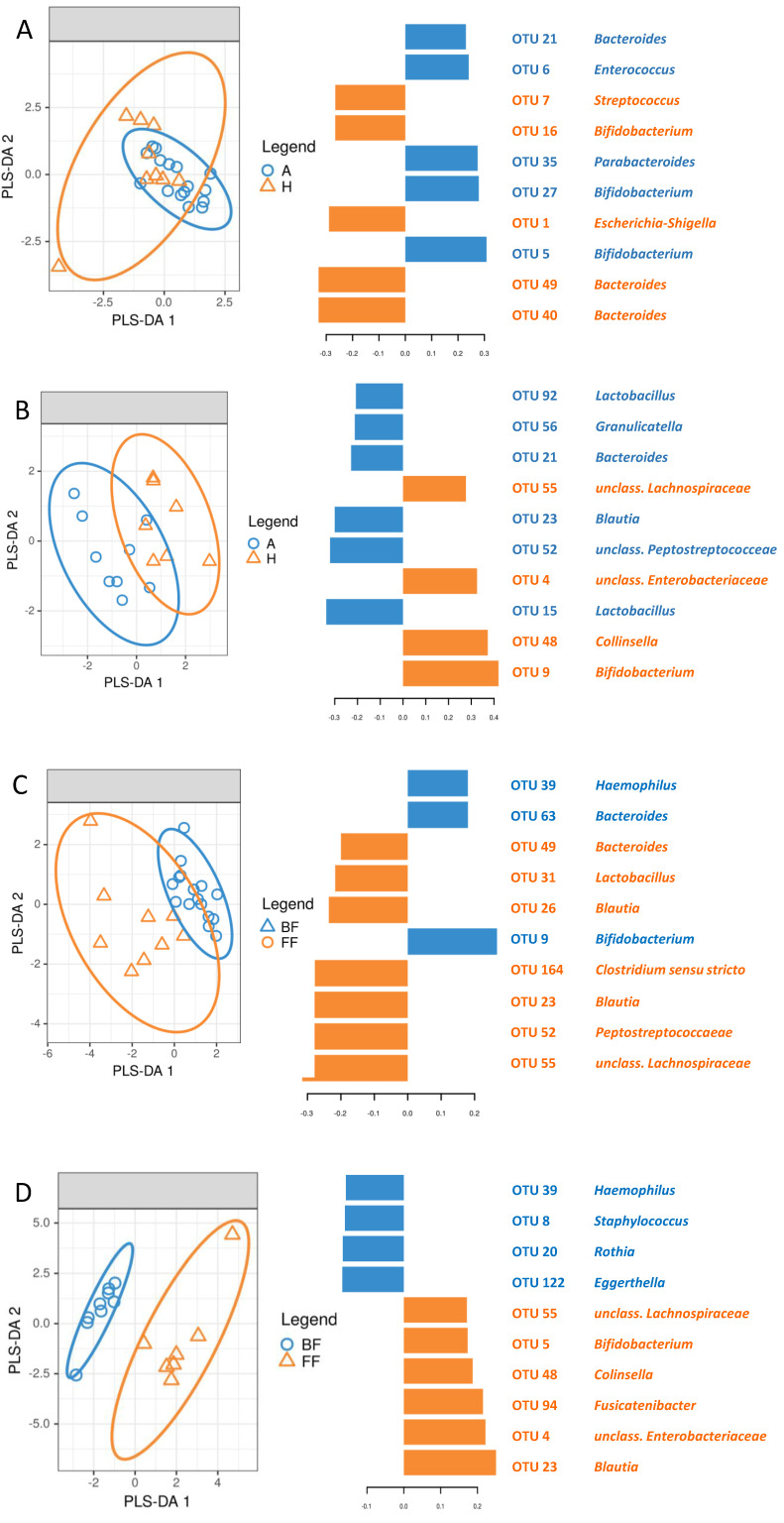
sPLS-DA analysis of fecal samples. (**A**) Breastfed infants; A—allergic, H—healthy; (**B**) Formula-fed infants; A—allergic, H—healthy; (**C**) Allergic infants; BF—breastfed, FF—formula-fed; (**D**) Healthy infants; BF—breastfed, FF—formula-fed.

The microbiome of the fecal samples from breastfed children was more similar to one another than in the case of formula-fed ones (Figure 5A,B), regardless of allergic status. OTUs differentiating allergic and healthy children are different in breastfed and formula-fed ones; only OTU21 (*Bacteroides*) was common for these two groups (Figure 5A,B).

The influence of breastfeeding on the fecal microbiome was more pronounced in healthy children (Figure 5C) and less conspicuous in allergic ones (Figure 5D). Apart from OTU39 (*Haemophilus*), which was characteristic for breastfed infants, and OTUs 23 (*Blautia*) and 55 (unclassified member of Lachnospiraceae), which were signature ones for formula-fed children, sets of OTUs differentiating breastfed and formula-fed patients were distinct in healthy and allergic groups.

**Figure 6:**
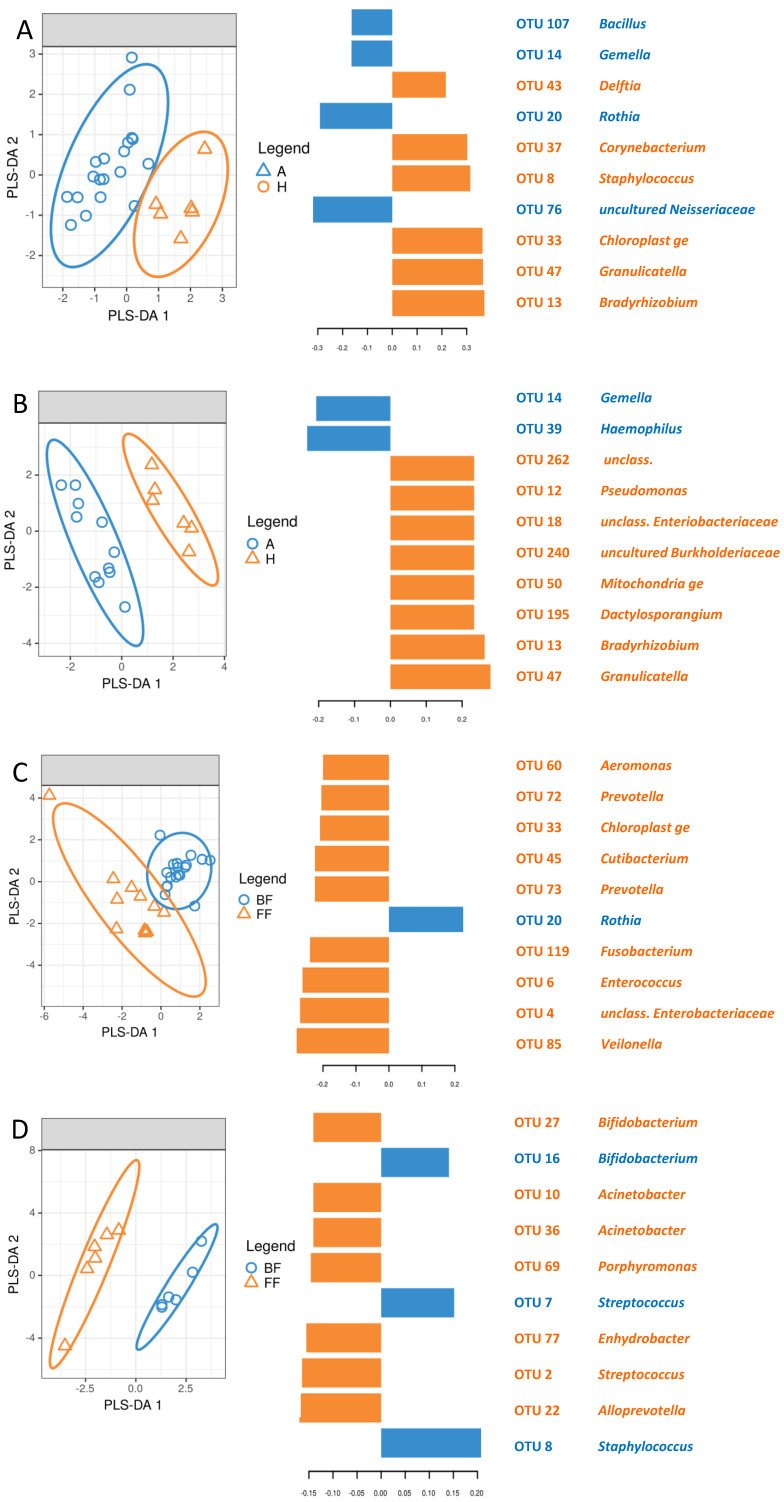
sPLS-DA analysis of skin samples. (**A**) Breastfed infants; A—allergic, H—healthy; (**B**) Formula-fed infants; A—allergic, H—healthy; (**C**) Allergic infants; BF—breastfed, FF—formula-fed; (**D**) Healthy infants; BF—breastfed, FF—formula-fed.

Similarly, the skin microbiome of healthy and allergic children differed less in the breastfed group (Figure 6A) than in the formula-fed one (Figure 6B). Signature OTUs were different in the two groups, apart from OTU14 (*Gemella*), which was characteristic for allergic infants, and OTU13 (*Bradyrhizobium*), which was characteristic for non-allergic patients. The breastfeeding influence was more pronounced in healthy children (Figure 6C) than in allergic ones (Figure 6D), and the differences were caused by distinct sets of signature OTUs.

### 3.6. Co-Occurrence of Bacteria in Milk and Feces as Well as Milk and Skin Depending on Allergic Status

There were positive correlations between milk and feces at the level of genus and OTU (Table 2). The number of reads classified as *Bifidobacterium* (ρ = 0.55, q = 0.0002), *Acinetobacter* (ρ = 0.35, q = 0.02) and *Enterobacter* (ρ = 0.41, q = 0.008) were correlated in the milk and feces samples when allergic and healthy patients were grouped together.

When allergic and healthy groups were analyzed separately, *Bifidobacterium* abundance was correlated in both groups (healthy ρ = 0.62, q = 0.03, allergic ρ = 0.51, q = 0.005), while *Acinetobacter* (ρ = 0.62, q = 0.03) and *Enterobacter* (ρ = 0.76, q = 0.003) were only correlated in healthy patients. At the OTU level, the sets of OTUs whose abundance was correlated in milk and feces were different in healthy and allergic patients, the only exception being OTU89 (*Streptococcus anginosus* ρ = 0.49, q = 0.07 in allergic, ρ = 1, q = 0 healthy) (Table 2).

*Acinetobacter* was the sole taxon whose abundance was correlated in milk and skin samples in whole group (ρ = 0.32, q = 0.04); however, the correlation was not significant in separated healthy and allergic groups. There were no significant correlations at the OTU level.

## 4. Discussion

The strengths and limitations of our study group were described earlier [28]. Additional strengths stem from the inclusion of milk; this is the first study analyzing the microbiomes of milk, skin and feces simultaneously. The limitations are related to (i) single timepoint sampling and (ii) non-homogeneity regarding clinical variables such as age and other factors that could impact allergy development. However, as we did not see any significant influence of these variables (given in [28]) on either alpha or beta diversity, we concluded that this non-homogeneity did not significantly impact our results. The infants' age is of the greatest concern here, but as stated above, there was no significant influence of this variable on alpha and beta diversity. Moreover, a majority of the children were between 12 and 16 weeks of age. Missing samples are another important limitation of our study. Their presence was caused by technical reasons: either the collection of certain samples was impossible (e.g., the patient was unable to deliver feces during a visit) or processing was hampered and there was no possibility of repeating an experiment. As we analyzed the sets of fecal, skin and milk samples separately, this limitation only concerns the correlations of bacterial abundance between sample types. It is likely that we missed certain correlations due to insufficient statistical power caused by the reduced number of samples.

Alpha diversity (measured as Shannon’s H’) patterns in our study were largely similar to those obtained by other groups. This includes the lack of differences between allergic and healthy children's mothers’ milk [13] as well as finding that breastfeeding decreases the difference between allergic and healthy children's feces microbiomes, lowering diversity in the former ones [5,12,13,15]. Lower diversity in the formula-fed infants' feces was observed only in cohorts consisting of premature children [23,24]. We observed lower richness on the skin of breastfed infants compared to that of formula-fed ones, while Yunge et al. showed no differences [1]. This fact might be explained by differences in the demographics (racial differences, ~30% of premature children) of the groups under study. A lower difference between breastfed allergic and healthy infants than between formula-fed ones supports the hypothesis of the beneficial influence of breastfeeding on the gut and skin microbiomes. No correlation was found between alpha diversity and clinical variables, which might be due to the low number of patients in our study group.

Bacterial communities in breastfed and formula-fed children were different both in the feces and skin samples, which is similar to previously published studies, e.g., Aparicio et al. [18], but there were no differences due to the allergic status. Similarly to the alpha diversity of feces and skin, the patterns of beta diversity observed in our study seem to back up the beneficial influence of breastfeeding hypothesis, as the ‘allergic microbiome’ (both in the guts and on the skin) is more similar to the ‘healthy’ one in breastfed infants than in the formula-fed ones. The lack of clinical variables influence on the community structure was probably caused by the low number of patients and great microbiome diversity.

In spite of the lack of significant differences in beta diversity (assessed as unweighted UniFrac distance), there were OTUs characteristic for healthy children’s mothers’ milk. Some OTUs were found exclusively or with greater frequency in the milk of healthy children’s mothers compared to the milk of allergic children’s mothers. Similar observations were reported earlier at the level of family [18]. The lack of overall difference might be caused by a large variability of communities. The differentially abundant OTUs came from a diverse set of phyla (Gammaproteobacteria, Bacteroidia and Fusobacteria) and apparently do not share common characteristics. Interestingly, two of the OTUs were classified as strains isolated from human sources [39,40], and one of the two (*Bacteroides ovatus*) is among the most common colonic bacteria [41] and was reported to cause elevated immune response in inflammatory bowel disease [39]. The taxonomic structure of bacterial communities in the milk of healthy and allergic infants’ mothers differed in only a few taxa, whose members were identified as signature OTUs in sPLS-DA, corroborating the latter results. A lower abundance of certain strains in samples coming from breastfed children might be explained by non-milk-derived strains being outcompeted by those that come from milk and by the growth promotion of the latter by milk-provided substances, such as HMOs. The differentially abundant bacteria more frequent in healthy patients and their mothers’ milk might be involved in protection against allergy. On the other hand, milk-transferred bacteria such as *Methylobacterium komagatae*, *Methylocapsa palsarum* and *Bacteroides vulgatus* are possibly related to an increased probability of developing celiac disease in childhood [19]. Similarly, the involvement of Egerthellaceae members [18] and lower microbiome diversity in the mother's milk [26] in allergy was implied. sPLS-DA signatures of healthy fecal and skin samples comprise different organisms, which might indicate that, in spite of presumably the same source of bacteria (i.e., milk), selection exerted by the immune system operates differently in these two compartments. Moreover, greater differences between the skin samples than between the fecal ones might indicate stronger selection due to a ‘longer route’ (i.e., microorganisms are first selected in the guts and then transferred to the skin, and on the way, they undergo selection as well). Stronger selection in the skin could also be caused by the external environment, particularly oxygen and UV light. The fact that we do not see a large overlap between milk, fecal and skin microbiomes suggests that low abundance organisms are selected, which indicates strong selection and a low share of stochasticity in community assembly [42].

In the case of fecal samples, the differences between communities of breastfed and formula-fed children were similar to those previously observed [2,5,10,20]. Woperis et al. [9] showed that the stool microbiome of infants at risk of developing allergies was shaped differently, depending on the feeding method (breastfeeding, formula supplemented with prebiotic or standard formula) and allergic symptoms. Our results reveal that the distinctions between the gut and skin microbiomes in breastfed and artificially fed children were different in the case of children with allergies and ones from the control group. The presented results support Sordillo’s hypothesis, which postulates that lower Clostridiales abundance is the factor conferring protection against allergy in breastfed children [15].

We found that both the skin and gut microbiomes of allergic children were more similar to that of healthy ones in the case of breastfed children. This fact supports the hypothesis that breastfeeding shifts the microbiome toward the ‘healthy’ state, which was found not only for allergies but also for other pathologies [6,9,23,25]. However, feeding mode-dependent differences in the community structure were greater in healthy patients than in allergic ones. This fact might be explained by the difference between an ‘allergic’ microbiome and a ‘healthy’ one [9,18,24].

For the first time, we observed the correlation of taxa abundance between milk and, fecal as well as between milk and skin samples. Moreover, in the case of the milk–feces correlation, the taxa were different for allergic and healthy patients, which points at a differently operating immune system. Our observation is not unexpected, as the influence of various body compartment microbiomes on one another, as well as differences between allergic and healthy gut and skin microbiomes, were frequently reported [1,15,18,43]. The relation between the gut microbiome and AD development was also postulated [14]. Importantly, the correlated taxa might be of particular importance in maintaining health or allergy development.

## 5. Conclusions

The mother’s milk microbiome influenced her child’s gut and skin microbiomes. Allergic and healthy patients differed both in skin and fecal microbiota.

Breastfeeding caused allergic children's fecal microbiomes to be more similar to those of healthy patients than in the case of formula-feeding.

Taxa whose abundance in milk and fecal samples was correlated might play an important role in protection against the development of allergy. Such strains might be used to formulate individualized probiotics taking into consideration not only the clinical picture but also the mode of feeding.

## Figures and Tables

**Table 1:** Comparison of bacterial taxa abundance between samples from healthy and allergic children and their mothers depending on allergy status and feeding mode.

Compared Groups	Material	Bacterial Taxa	Incidence	q
Breastfed: allergic vs healthy	milk	*OTU3 (Pseudomonas peli)* *OTU40 (Bacteroides ovatus)* *OTU93 (Leptotrichia wadei)*	healthy only healthy only healthy > allergic	0.03 0.03 0.04
	feces	*OTU30 (Serratia marcescens)* *OTU68 (Lactococcus lactis)* *OTU35 (Parabacteroides)*	healthy only healthy only allergic only	0.03 0.03 0.04
	skin	-		
Allergic: breastfed vs formula-fed	feces	*Blautia* *Eschericha-Shigella* *OTU1 (Escherichia-Shigella)* *OTU52 (Peptostreptococcaceae)* *OTU55 (Lachnospiraceae)*	formula-fed > breastfed	0.025 0.04 0.04 0.02 0.02
	skin	*Enterobacteriales* *Pseudomonadales* *Enterobacteriaceae* *Moraxellaceae*	formula-fed > breastfed	0.02 0.049 0.02 0.04
Healthy: breastfed vs formula-fed	feces	*OTU23 Blautia*	formula-fed > breastfed	0.04
	skin	*Bacteroidales*	formula-fed > breastfed	0.02

q: *p*-value corrected for multiple comparisons with Benjamini-Hochberg method.

**Table 2:** Co-occurrence of OTUs in milk and feces.

Allergic Group				Healthy Group			
OTU		*rho*	q	OTU		*rho*	q
2	*Steptococcus*	0.44	0.02	5	*Bifidobacterium longum*	0.64	0.02
20	*Rothia mucillaginosa*	0.46	0.02	10	*Acinetobacter johnsoni*	0.65	0.02
21	*Bacteroides*	0.37	0.05	16	*Bifidobacterium scardivii*	0.74	0.004
27	*Bifidobacterium bifidum*	0.46	0.02	25	*Acinetobacter*	0.61	0.03
31	*Lactobacillus gasseri*	0.48	0.008	30	*Serratia marcescens*	0.61	0.03
48	*Colinsella aerofaciens*	0.53	0.003	32	*Haemophilus haemolyticus*	0.61	0.03
62	*Agrobacterium fabrum*	0.42	0.02	89	*Streptococcus anginosus*	1	0
81	*Atopobium parvulum*	0.62	0.0001				
89	*Streptococcus anginosus*	0.49	0.07				
95	*Lactobacillus oris*	0.53	0.003				

q: *p*-value corrected for multiple comparisons with Benjamini-Hochberg method.

## Data Availability

Sequence data generated during this project are available in the SRA database under BioProject no. PRJNA657878.
